# Complete Genome Sequence of Kocuria indica CE7, Isolated from Human Skin

**DOI:** 10.1128/MRA.00607-19

**Published:** 2019-07-11

**Authors:** Kyoung Lee, Munkhtsatsral Ganzorig, Ji Yoon Jung, Sachin Kumar Badaya, Jae Yun Lim

**Affiliations:** a Department of Bio Health Science, Changwon National University, Changwon, South Korea; b Amity Institute of Microbial Biotechnology, Amity University, Noida, India; c School of Systems Biomedical Science, Soongsil University, Seoul, South Korea; University of Rochester School of Medicine and Dentistry

## Abstract

Here, we report the complete genome sequence of Kocuria indica CE7, isolated from human skin. This strain possesses a 2,809-kbp chromosome and a 32-kbp plasmid with 2,507 coding sequences. In particular, the genome contains multiple genes potentially involved in adaptations in pH homeostasis and salt tolerance.

## ANNOUNCEMENT

Kocuria indica is a high-GC-content Gram-positive aerobic nonmotile and coccus-type bacterium belonging to the family *Micrococcaceae* in the phylum *Actinobacteria*. *K. indica* NIO-1021^T^ was first isolated from a sediment sample from Chorao Island, India (1). *K. indica* strain NIO-1021^T^ is known to grow in the presence of 0 to 15% NaCl and in the pH range of 4.0 to 10. In the comparative analysis of 16S rRNA gene sequences, *K. indica* forms a monophyletic clade with Kocuria marina with 100% bootstrap values compared to 17 other species (1). Two incomplete genome sequences each of *K. indica* and *K. marina* are currently available in the NCBI database. Here, we report the complete genome sequence of *K. indica* CE7, an isolate from human skin.

*K. indica* CE7 (KCTC 49187) was isolated from the forehead of a male college student using swab sampling and direct streaking onto Difco nutrient agar. The agar medium was incubated for 3 days under aerobic conditions at 28°C. Ethical approval for subject sampling was granted by the institutional review board of Changwon National University. The phenol extraction method (2) was used to extract total DNA from *K. indica* CE7 cells, which were cultured for 2 days in a shaking incubator (140 rpm) for 48 h at 28°C. The genomic SMRTbell library using a total of 5 μg of purified DNA was constructed with the SMRTbell template prep kit v1.0 (product number 100-259-100) following the manufacturer’s instructions (Pacific Biosciences). The fragments of the template smaller than 20 kbp were removed using the BluePippin size selection system, and the constructed library was validated using an Agilent 2100 bioanalyzer. The SMRTbell library was sequenced using 1 single-molecule real-time (SMRT) cell using C4 chemistry, and 240-minute movies were captured for each SMRT cell using the MagBead OneCellPerWell v1 protocol (Pacific Biosciences) of the PacBio RS II sequencing platform (DNA Link, Seoul, South Korea). *De novo* assembly was conducted according to the Hierarchical Genome Assembly Process (HGAP) v2.3 workflow, including consensus polishing with Quiver (3). Control filtering (RemoveControlReads.1.xml) and a seed read length of 13,000 bp were used as options for the HGAP workflow. After filtering the subreads, a pool of 1,312,238,763 bp from 168,966 reads with an *N*_50_ value of 2,820,133 bp was produced, yielding a 124-fold genome coverage. In order to check if the contigs were circular, bioinformatics programs such as NUCmer and MUMmer v3.5 (4) were used to identify overlapped sequences of both ends for manual genome closure. Gene predictions and annotations were provided by NCBI using the NCBI Prokaryotic Genome Annotation Pipeline (5).

The CE7 assembly consists of a circular chromosome of 2,809,846 bp (68.7% GC content) and a circular plasmid (pCE7) of 32,758 bp (64.5% GC content). The chromosome contains 2,472 coding sequences (CDSs), 3 copies of rRNA genes (5S, 16S, and 23S), 46 coding regions of tRNAs, 2 noncoding RNAs (ncRNAs), and 1 transfer-messenger RNA (tmRNA). Compared to the *K. indica* NIO-1021^T^ reference genome, the chromosome sequence of *K. indica* CE7 showed 97.10% (82.82% coverage) average nucleotide identity based on BLAST average nucleotide identity (ANIb), as determined by the JSpeciesWS server (6). The pCE7 plasmid carries 35 CDSs, including 17 hypothetical proteins. Since *K. indica* has been known to be salt tolerant and grows in a wide range of pH values, the genes related to the niche were sought in the genome. The CE7 genome contains genes encoding two NhaA and one Mrp protein complex for Na^+^:H^+^ antiporters, a Ca^2+^:H^+^ antiporter, and a cation:H^+^ antiporter, which are known to play critical roles in alkaline pH homeostasis in cells (7). In addition, the CE7 genome possesses the genes encoding trehalose biosynthesis, the amino acid:cation symporter family proteins, the Na^+^:solute symporter, and the K^+^ uptake system, which confer resistance to osmotic stress.

### Data availability.

The complete genome sequences of the chromosome and plasmid pCE7 of the *K. indica* CE7 strain were deposited in GenBank under the accession numbers CP035504 and CP035505, respectively. The raw sequencing data have been deposited in the SRA under the accession number PRJNA517384.
